# Long noncoding RNA UCA1 from hypoxia-conditioned hMSC-derived exosomes: a novel molecular target for cardioprotection through miR-873-5p/XIAP axis

**DOI:** 10.1038/s41419-020-02783-5

**Published:** 2020-08-10

**Authors:** Ling Sun, Wenwu Zhu, Pengcheng Zhao, Qingjie Wang, Baohan Fan, Yeqian Zhu, Yao Lu, Qiushi Chen, Jian Zhang, Fengxiang Zhang

**Affiliations:** 1 Section of Pacing and Electrophysiology, Division of Cardiology, The First Affiliated Hospital with Nanjing Medical University, Nanjing, China; 2 Department of Cardiology, The Affiliated Changzhou No. 2 People’s Hospital of Nanjing Medical University, Changzhou, China

**Keywords:** Long non-coding RNAs, Mesenchymal stem cells

## Abstract

Exosomes (Exo) secreted from mesenchymal stem cells (hMSCs) are protective against myocardial injury. The purpose of the study was to investigate the role and mechanisms by which exosomes promote cardiomyocyte survival and function following myocardial infarction (MI). hMSCs were cultured under hypoxic and normoxic conditions. Hypoxia-conditioned hMSC-derived exosomes (Hypo-Exo) and normoxic-conditioned hMSC-derived exosomes (Nor-Exo) were collected and intramyocardially injected into rats with MI. The therapeutic effects of Hypo-Exo and Nor-Exo were evaluated after 4 weeks. Quantitative real-time PCR (qRT-PCR) was used to detect the expression of candidate long noncoding RNA urothelial carcinoma associated 1 (lncRNA-UCA1) in Nor-Exo and Hypo-Exo. Intramyocardial injection of lncRNA-UCA1-knockdown-Hypo-Exo in a rat model of MI was then performed and the cardiac function was characterized. The target and downstream of the molecular mechanism lncRNA-UCA1 was disclosed by luciferase reporter assays and western blot. Circulating exosomal lncRNA-UCA1 level in AMI patients and healthy volunteers was assessed. We found that (1) hMSC exosomal (from hypoxic and normoxic conditions) cardioprotection in vitro and in vivo correlated with the presence of encapsulated lncRNA-UCA1 in exosomes; (2) lncRNA-UCA1 targeted miR-873 via sponging, reducing the latter’s suppressive effects on its target XIAP, and this translated into AMPK phosphorylation and increased level of the antiapoptotic protein BCL2; and (3) plasma derived from patients with AMI contained exosomes enriched with the lncRNA-UCA1, unlike that from normal subjects. This study demonstrates that Hypo-Exo lncRNA-UCA1 plays a cardioprotective role via the miR-873-5p/XIAP axis and circulating exosomal lncRNA-UCA1 may be a promising novel biomarker for the diagnosis of AMI.

## Introduction

Ischemic heart disease is a leading cause of death^1^ of which acute myocardial infarction (AMI) is an important cause, leading to a poor prognosis^2,3^. Coronary artery occlusion leads to hypoxia with consequent death of cardiomyocyte^4^. Currently, stent insertion and thrombolysis are the main treatment methods^5,6^. Nonetheless, they can be associated with reperfusion injury, including myocardial cell death and microvascular injury^7^. An effective alternative therapy for myocardial infarction (MI) that can reduce cardiomyocyte loss is therefore urgently required.

Transplantation of stem cells has been reported to promote cardiac function after MI^8^. Recently, several studies have confirmed this cardioprotective function of stem cells, largely achieved by secretion of paracrine factors including exosomes (Exo)^9^. Exo are small (30–200 nm) endogenous membrane vesicles secreted by most cells^10^ and transmit a variety of signaling molecules including proteins, microRNAs (miRNAs), and long noncoding RNAs (lncRNAs)^11^ as well as mediating cell-to-cell communication and crosstalk between organs. In addition, compared with stem cell transplantation, Exo have the advantages of a low tumorigenic potential, minimal immunogenicity, and do not lead to teratoma formation. They are therefore considered to be a promising cell-free therapy for cardiac repair^10^.

A recent study suggested that mesenchymal stromal cell-derived Exo could attenuate injury through miR-182-regulated macrophage polarization^12^. Another study demonstrated that injection of hypoxic-conditioned human mesenchymal stem cell (hMSC)-derived Exo (Hypo-Exo) in MI mice improved the therapeutic effects of normoxia-conditioned hMSC-derived Exo (Nor-Exo) via miR-125b-5p^12,13^. Nonetheless few studies have reported the therapeutic effects of MSC-derived Exo in AMI via mediating lncRNA^14^. In the current study, we investigated the protective role of Hypo-Exo using a rat model of MI. We established that Hypo-Exo is enriched with lncRNA urothelial carcinoma associated 1 (lncRNA-UCA1) that exhibits an antiapoptotic effect in vivo and in vitro. We revealed that knockdown of lncRNA-UCA1 in Hypo-Exo significantly increased the infarct area and promoted cardiomyocyte apoptosis. In addition, circulating exosomal lncRNA-UCA1 level in AMI patients has the potential to serve as a diagnostic biomarker.

## Materials and methods

### Ethical considerations

The present study was conducted in accordance with the principles of the Declaration of Helsinki and approved by the Ethics Committee of the First Affiliated Hospital and Changzhou No. 2 People’s Hospital of Nanjing Medical University.

### Patients

Blood samples were obtained from 26 patients with confirmed AMI (from the cardiac care unit) and 26 healthy volunteers (from the health examination center) at Changzhou No. 2 People’s Hospital of Nanjing Medical University. Informed consent was obtained from all study subjects.

### Cell culture, hypoxia exposure, and transfection

All cells were incubated at 37 °C in 5% CO_2_ and 21% O_2_ under normal condition. hMSCs from healthy adults were purchased from Cambrex BioScience and cultured in α-minimal essential medium (α-MEM) with 10% fetal bovine serum (FBS). The surface marker of hMSCs was determined using flow cytometry. In the hypoxic experiments, hMSCs were transferred into a hypoxic chamber (Thermo Forma, USA) with 1% O_2_, 5% CO_2_, and 94% N_2_ for 48 and 12 h, and Exo were isolated. H9c2 cardiomyoblasts (American Type Culture Collection) were cultured in Dulbecco’s modified Eagle’s medium (DMEM) (Gibco, USA) with 10% FBS. In the hypoxic and serum deprivation (H/SD) experiments, H9c2 cells were cultured with DMEM with no glucose (11966025, Gibco, USA) in 94% N_2_, 5% CO_2_, and 1% O_2_ for 12 h.

Transfection of lncRNA silencer (200 nmol/l), miRNA mimics (100 nmol/l), miRNA inhibitor (200 nmol/l), and their negative controls (100–200 nmol/l) was carried out using riboFECT^TM^ CP Reagent (Ribobio, China) according to the manufacturer’s instructions.

### Exo isolation and characterization

Human blood samples were collected in an ethylene diamine tetraacetic acid anticoagulant tube and centrifuged immediately (3000 × *g*, 15 min). Supernatant (plasma) was obtained and thawed on ice. Ribo^TM^ Exosome Isolation Reagent (a precipitation reagent for plasma or serum, C10110-2, Ribobio, China) was used for Exo isolation according to the manufacturer’s instructions. In brief, plasma samples were centrifuged at 2000 × *g* for 20 min to remove cells and cell debris. Clarified plasma samples (3 ml) were then transferred to a clean tube containing 1 ml Ribo Exosome Isolation Reagent. The mixtures were incubated overnight at 4 °C and then centrifuged at 15,000 × *g* for 2 min. Supernatant was aspirated and the Exo resuspended in 100 μl 1× phosphate-buffered saline (PBS).

hMSCs (1 × 10^6^) were grown in a cell culture flask (T25, Corning, USA) to 80% confluence. Cells were then washed once with PBS and treated with 5 ml Exo-free FBS (Gibco) for 2 days. Cell culture medium (5 ml) was harvested in a 15-ml conical tube and centrifuged at 1500 × *g* for 30 min to remove cells and debris. Cell-free media was transferred into another 15 ml conical tube containing 2 ml Ribo^TM^ Exosome Isolation Reagent (for cell culture media, C10130-2, Ribobio, China). Similarly, these mixtures were incubated overnight at 4 °C and then centrifuged at 2000 × *g* for 30 min. The supernatant was aspirated and the Exo resuspended in 100 μl PBS.

A BCA Protein Assay Kit (Thermo Fishier Scientific, USA) was used to measure exosomal proteins. The surface markers of Exo were detected using western blotting with anti-TSG101, CD63, and CD81 antibodies (Abcam, UK). These Exo were fixed with 1% glutaraldehyde, applied on a carbon-coated copper grid, and then stained with 1% phosphotungstic acid. The samples were tested using a JEM-2100 transmission electron microscope (TEM; JEOL, Japan). Size distribution and concentration of particles was evaluated by nanoparticle tracking analysis (NTA) using ZetaView PMX 110 (Particle Metrix, Germany)^15^. Brownian motion of particles was tracked and recorded by laser scattering microscopy. The diameters of the particles and size distribution data were analyzed using the Stokes–Einstein equation.

### Exo uptake assay

To determine whether Exo could be absorbed by H9c2 cells, Exo were labeled with a red fluorescent dye (Dil, Invitrogen). Briefly, 1 µM Dil was incubated with 100 µg Exo for 15 min. To remove excess dye, 3 ml PBS and Exosome Isolation Reagent were added, and the mixture was centrifuged at 1500 × *g* for 30 min. Supernatant was then aspirated, and the Dil-labeled Exo were resuspended in 100 μl PBS. The Dil-labeled Exo were co-cultured with H9c2 cells at 37 °C. The cells were subsequently washed with PBS and fixed with 4% paraformaldehyde for 15 min. Fixed cells were washed with PBS and nuclei stained with 4′,6-diamidino-2-phenylindole (DAPI, 0.5 μg/ml; Invitrogen). A confocal microscope (Carl Zeiss, Germany) was used to observe the red signals in cells.

### Cell viability assay

Cell viability was measured using the Cell Counting Kit-8 (CCK-8, Dojindo, Japan). Briefly, H9c2 cells (2000 cells/well) were seeded in 96-well plates and cultured with PBS or Exo (200 μg/ml) for 10 h. Ten microliters of CCK-8 solution was added to each well. After 2 h, absorbance was measured at 450 nm using a Synergy 2 microplate reader (Bioteck, USA). The percentage of viable cells was calculated with the control cells set as 100%.

### Apoptosis assays

Flow cytometry (KeyGEN Biotech, China) was used to assess cell apoptosis. H9c2 cells were cultured overnight at a seeding density of 1 × 10^5^/6-well tissue culture plates and treated with Exo or PBS before being subjected to hypoxia. To quantify the apoptotic cells, cells were washed with PBS and stained using an Annexin V-Fluorescein Isothiocyanate and Propidium Iodide Apoptosis Kit (KeyGen Biotech, China). The apoptotic cells were analyzed using the Flowjo Software version 10.0 (Tree Star, USA). TdT-mediated dUTP Nick-End Labeling (TUNEL) Apoptosis Detection Kit (Roche, USA) was also used to determine cell or tissue apoptosis according to the manufacturer’s instructions. The percentage of apoptotic nuclei was calculated by dividing the total number of TUNEL-stained nuclei by the total number of TUNEL-positive nuclei.

### Quantitative real-time PCR (qRT-PCR)

qRT-PCR was performed using the 7900HT Real-Time PCR Detection System (Thermo Fisher Scientific, USA). Reactions were performed in triplicate with each sample. Briefly, the total cellular and exosomal RNA was extracted using Trizol reagent (Life Technologies, USA). For quantification of lncRNA-UCA1 or X-linked inhibitor of apoptosis protein (XIAP) mRNA, first-strand cDNA was synthesized with random primers using a PrimeScript™RT Reagent Kit with gDNA Eraser (Takara, Japan). Stem-loop qRT-PCR was performed using a FastStart Essential DNA Green Master (Roche, USA). The cellular expression of lncRNA-UCA1 and XIAP mRNA was normalized to that of glyceraldehyde 3-phosphate dehydrogenase (GAPDH)^16^. The exosomal level of lncRNA-UCA1 was normalized to that of cel-miR-39 (C39)^17,18^. For quantification of miR-873-5p, cDNA was synthesized with an miRNA First-Strand cDNA Synthesis Kit (by stem-loop) (Vazyme Biotech, China). AceQ qPCR SYBR Green Master Mix (Vazyme Biotech, China) was then used for real-time PCR. The cellular miR-873-5p expression was normalized to U6. The relative expression was calculated using the following equation: relative gene expression= 2^−(ΔCtsample − ΔCtcontrol)^. The primers are listed in Supplementary Table 1.

### Western blot

Cells were lysed on ice for 30 min with lysis buffer. Total cell protein concentration was determined using a BCA Protein Assay Kit. The total protein (30 μg) was resolved using sodium dodecyl sulfate-polyacrylamide gel electrophoresis (Invitrogen) and transferred to a polyvinylidene fluoride membrane (Roche). Membranes were blocked with 5% bovine serum albumin in TBS-Tween (0.1%) and incubated against the desired antibodies. The primary antibodies BAX (5023, Cell Signaling Technology, USA), BCL-2 (ab196495, abcam, USA), p53 (21083, Signalway Antibody, USA), AMP-activated protein kinase (AMPK, 5831, Cell Signaling Technology), phosphorylation of AMPK (p-AMPK, 500815, Cell Signaling Technology), XIAP (14334, Cell Signaling Technology), GAPDH (5174, Cell Signaling Technology), TSG101 (14497, Proteintech, United States), CD63 (25682, Proteintech), CD81 (66866, Proteintech), and horseradish peroxidase-conjugated secondary antibody (Santa Cruz) were used. Bands were visualized using enhanced chemiluminescence reagents and analyzed using a gel documentation system (Bio-Rad Gel Doc1000 and Multi-Analyst version 1.1).

### Exosomal lncRNA-UCA1 knockdown

For depletion of lncRNA-UCA1, hMSCs were stably transfected with GV248/UCA1 short hairpin RNA (shRNA) or GV248/control shRNA plasmid (GeneChem, China). The three-pooled shRNA sequences that yielded the greatest knockdown are listed in Supplementary Table 2. Seventy-two hours later, hMSCs were cultured in α-MEM under hypoxic conditions for 48 h. The Exo derived from hMSCs were then isolated, as described previously.

### Luciferase assay

The fragment of lncRNA-UCA1 3′ untranslated region (UTR) cDNA containing the miR-873-5p-binding site was amplified by PCR. The amplified products were cloned in PGL3 vector (Promega, USA) immediately downstream of the stop codon of the luciferase gene. The mutant of lncRNA-UCA1 was constructed using the Takara MutanBEST Kit (Takara, Japan). For the luciferase assay, HEK-293T cells in 24-well plates were transfected with 200 ng/well luciferase reporter constructs, 400 ng/well miR-873-5p mimic, or mimic control using Lipofectamine 2000. SV-Renilla luciferase plasmids (5 ng/well) served as the internal control. Cells were harvested at 24 h after transfection, and the luciferase activity detected using the Dual Luciferase Reporter Assay Kit (Promega, USA) as per the manufacturer’s instructions. Firefly luciferase activities were normalized to Renilla luciferase activity.

### Animal experiments

Male Sprague-Dawley rats (8 weeks of age, *n* = 6 for each group) were obtained from the experimental animal center of Nanjing Medical University. All surgical procedures and animal care protocols were performed in accordance with the Guide for the Care and Use of Laboratory Animals published by the U.S. National Institutes of Health. Animal protocols were performed with approval of the Experimental Animals Ethics Committee of the First Affiliated Hospital of Nanjing Medical University (No. IACUC-1905024). The animals were randomly divided into different treatment groups. All surgeries and subsequent analyses were blinded for intervention.

### MI protocol, Exo delivery, and myocardial injury assessment

All rats were anesthetized via intraperitoneal injection of 100 mg/kg ketamine combined with 10 mg/kg xylazine and then intubated and ventilated. The left anterior descending (LAD) coronary was ligated to induce MI^14^. One hundred microliters of Exo (1 µg/µl) or 100 µl PBS were injected into the border zone of the infarcted heart at three locations. To evaluate cardiac function after treatment, transthoracic two-dimensional (2D) M-mode echocardiography was performed using a Vevo 2000 high-resolution micro-imaging system (Visual Sonic) 2 and 4 weeks after MI. The parameters of heart function were recorded in 2D and M-mode from the parasternal long-axis view at the papillary muscle level. Left ventricular end-diastolic dimension (LVEDD) and left ventricular end-systolic dimension (LVESD) were measured in at least three consecutive cardiac cycles. Left ventricular ejection fraction (LVEF) and left ventricular fractional shortening (LVFS) were used to evaluate the systolic function of the hearts. LVEF was calculated as [(LVEDD)^3^ − (LVESD)^3^]/(LVEDD)^3^] × 100% and LVFS was calculated as [(LVEDD − LVESD)/LVEDD] × 100%. All measurements were made by an independent blinded sonographer. Finally, rats were sacrificed by intraperitoneal administration of pentobarbital.

To evaluate whether Exo could be absorbed by the myocardium, Dil-labeled Exo were injected in rats with MI. Six hours later, rats were sacrificed. The hearts were dehydrated and frozen, and the ventricular papillary muscle was sliced into 6-μm-thick cryosections. The heart sections were immunofluorescent stained with α-actin and then dyed with DAPI. Internalization of Exo into myocardium was observed by fluorescence microscope.

Infarct size was measured by Masson staining; myocardial apoptosis was determined by TUNEL staining using an In Situ Cell Death Detection Kit (Roche, USA).

### Statistical analysis

Continuous and categorical variables are described as mean ± SEM and percentages (%), respectively. For continuous variables, Student’s *t* test (normal distribution data) or Mann–Whitney *U* test (abnormal distribution data) were used. One-way analysis of variance (three groups) followed by Bonferroni’s correction, if needed, were performed. For categorical variables, chi-squared test was used. Receiver operating characteristic (ROC) curve analysis was performed to discriminate AMI patients from healthy controls. The area under the ROC curve (AUC) was measured. All statistical tests were performed with the GraphPad Prism software version 5.0, and *P* < 0.05 (two-sided) was considered statistically significant.

## Results

### Identification of Exo derived from hMSCs cultured under normoxic and hypoxic conditions

**Fig. 1:**
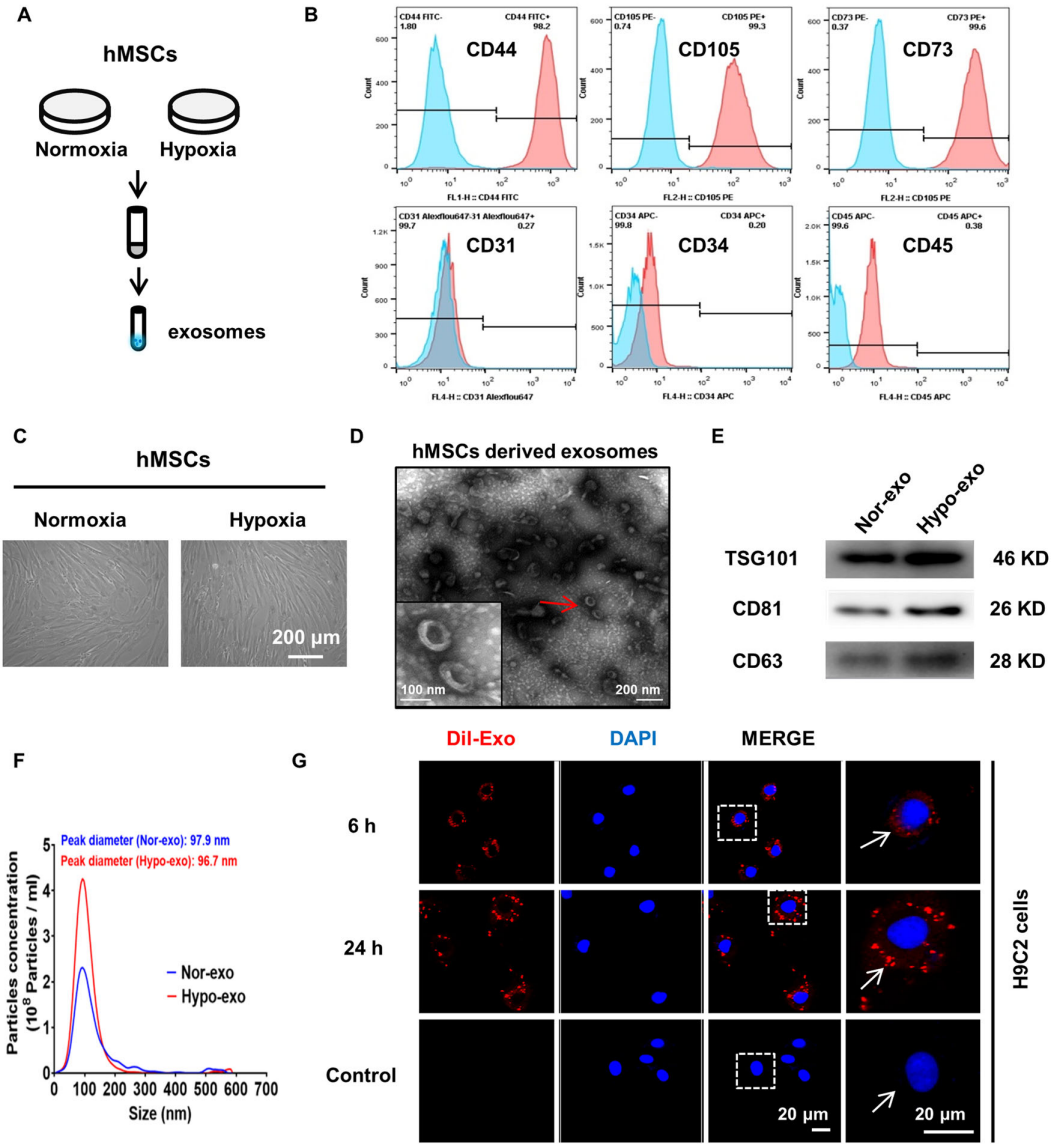
Characterization of exosomes derived from hMSCs cultured under normoxic and hypoxic conditions. **a** Exosomes were extracted from mesenchymal stem cell (hMSC) supernatants under normal and hypoxic conditions. **b** Surface markers of hMSCs detected by flow cytometry: positive for CD44, CD105, and CD73; negative for CD31, CD34, and CD45. **c** Representative image showing the morphology of normoxic hypoxic hMSCs under light microscopy. **d** Electron micrograph-analyzed hMSC-derived exosomes. The red arrow indicates exosomes. Scale bar: 100 nm. **e** Western blot showing the protein level of TSG101, CD63, and CD81 in normoxia-conditioned hMSC-derived Exo (Nor-Exo) and hypoxia-conditioned hMSC-derived Exo (Hypo-Exo), respectively. **f** Size distribution of Nor-Exo and Hypo-Exo determined by nanoparticle tracking analysis (NTA). *X* axis represents size of exosomes. *Y* axis represents the particle concentration in 1 ml PBS (before dilution). **g** H9c2 cardiomyocytes were cultured in the presence or absence (control) of Dil-labeled exosomes (red) at 37 °C for 6 and 24 h. The nucleus was stained with DAPI (blue). The white arrow indicates Dil-labeled exosomes absorbed by exosomes.

hMSCs were cultured under normoxic or hypoxic conditions. Supernatants were collected and Exo were extracted (Fig. 1a, c). Flow cytometry revealed that hMSCs were positive for CD44, CD73, and CD105 and negative for CD31, CD34, and CD45 (Fig. 1b). As shown in Fig. 1d, Exo showed typical lipid bilayer membrane-encapsulated nanoparticles. Figure 1e demonstrates that the proteins of TSG101, CD81, and CD63 were positively expressed in normoxic and hypoxic Exo. NTA showed that the peak diameter of normoxic and hypoxic Exo was 96.7 and 97.9 nm, respectively (Fig. 1f). The protein concentration of Hypo-Exo was increased significantly compared with that of Nor-Exo. In addition, NTA analysis revealed a similar trend, indicating an increase in the number of hMSC-derived Exo under hypoxic conditions. There were no significant differences in average size between Hypo-Exo and Nor-Exo (Supplementary Table 3).

The Exo were labeled with Dil dye and co-cultured with H9c2 cells for 6 and 24 h. Confocal microscopy showed that Dil-labeled Exo could be observed around the nucleus and appeared in a time-dependent manner (Fig. 1g). Collectively, these results indicate that normoxic and hypoxic hMSCs could secrete Exo with common exosomal features and these Exo could be absorbed by H9c2 cells.

### Hypo-Exo exerted better cardioprotection against myocardial injury than Nor-Exo in vitro and in vivo

**Fig. 2:**
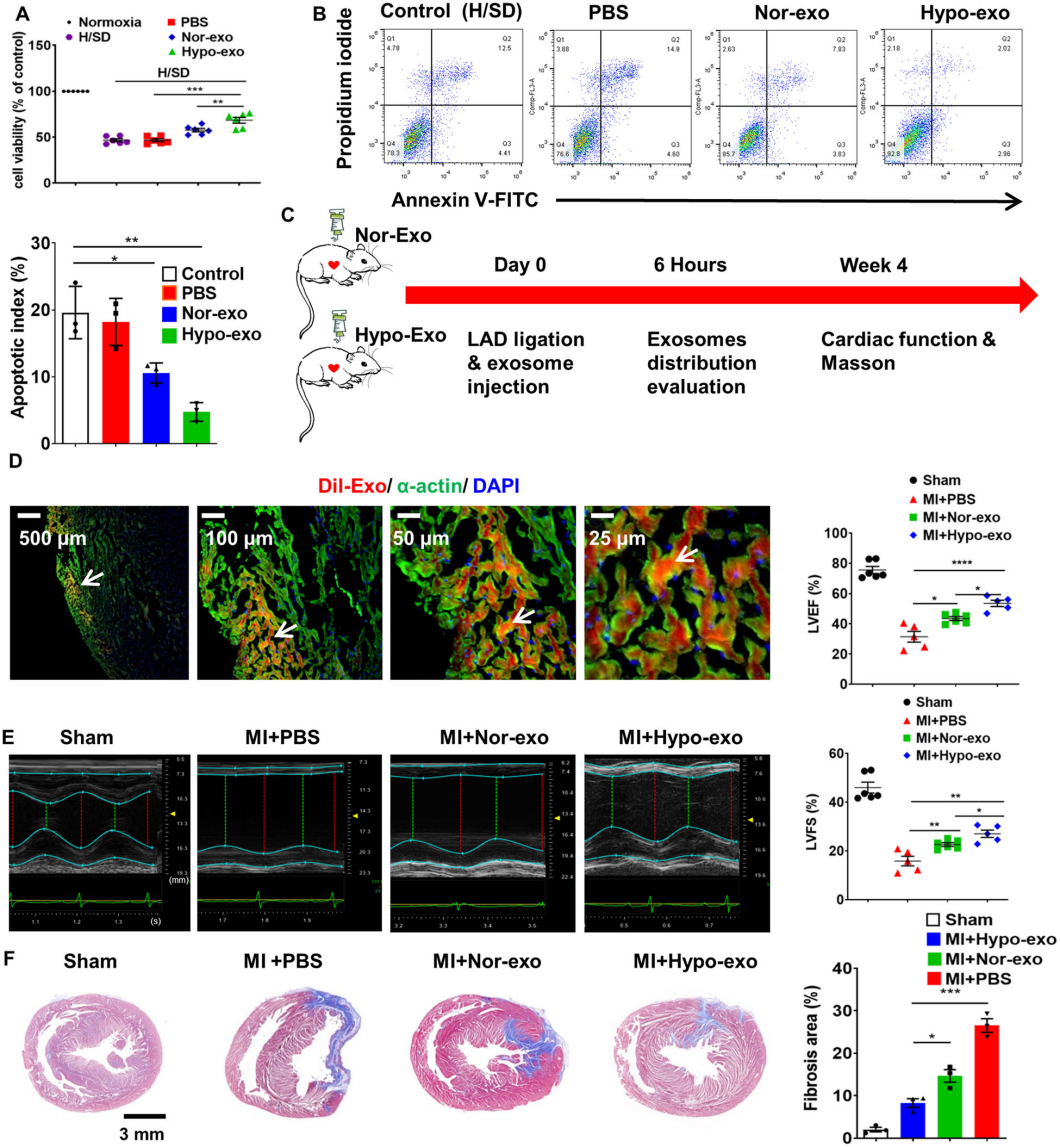
Hypo-Exo exerts better cardioprotective effects against myocardial injury than Nor-Exo in vitro and in vivo. **a** Hypo-Exo improved cell viability in H9c2 cells under normal and H/SD conditions (*n* = 6). **b** Hypo-Exo decreased apoptosis in H9c2 cells under H/SD conditions (*n* = 3). **c** Flowchart of in vivo experimental design. **d** Dil-labeled exosomes were injected into the infarcted heart of rats for 6 h (50 μg exosomes per rat). Representative images of post-MI heart sections stained with Dil-labeled Exo (red), α-actin (green), and DAPI (blue). The white arrow indicates that exosomes were absorbed by cardiomyocytes. **e** Representative echocardiographic images showing heart function among the different groups on the 28th day following MI. Quantitative analysis of left ventricular ejection fraction (LVEF) and left ventricular fraction shortening (LVFS) among the different groups (*n* = 6 for sham group, *n* = 5 for MI + PBS group, *n* = 6 for MI + Nor-Exo group, *n* = 5 for MI + Hypo-Exo group). **f** Masson’s trichrome-stained myocardial sections on the 28th day following MI in rats treated with sham, PBS, Nor-Exo, and Hypo-Exo. Scar tissue and viable myocardium are identified in blue and red, respectively (*n* = 3). Data are presented as mean ± SEM. Statistical analysis was performed with one-way ANOVA followed by Bonferroni’s correction. *P < 0.05, **P < 0.01, ***P < 0.001, ****P < 0.0001.

Nor-Exo, Hypo-Exo, and PBS were co-cultured with H9c2 cells under normal and H/SD conditions. Cell viability was significantly increased in the Nor-Exo and Hypo-Exo groups compared with the PBS group. Moreover, Hypo-Exo had an even better performance than Nor-Exo (Fig. 2a). Hypo-Exo and Nor-Exo also suppressed apoptosis when co-cultured with H9c2 cells under H/SD conditions (Fig. 2b and Supplementary Fig. 1). A rat model of AMI, achieved by permanent LAD artery ligation, was used to determine the cardioprotective effects of hMSC-derived Exo in vivo. Hypo-Exo, Nor-Exo (100 µl, 1 µg/µl), or 100 µl PBS were intramyocardially injected into rats at the time of MI (Fig. 2c). To observe whether Exo could be absorbed by cardiomyocytes, Dil-labeled Exo was intramyocardially injected. Immunofluorescent staining for cardiomyocyte-specific (α-actin) antigens was performed. Dil-labeled Exo were co-localized with cardiomyocytes 6 h after injection, suggesting an efficient in vivo uptake of the Exo by cardiomyocytes (Fig. 2d). LVEF and LVFS were significantly improved in both Nor-Exo- and Hypo-Exo-injected animals compared with those injected with PBS. LVEF and LVFS were higher in the Hypo-Exo group than those in the Nor-Exo group (Fig. 2e). Quantification of the infarcted area 4 h post-MI indicated that Hypo-Exo-injected rats had a smaller fibrotic area than PBS-injected rats (8.27% ± 1.78% vs. 26.53% ± 2.75%, *P* < 0.001) and Nor-Exo-injected rats (14.66% ± 2.57%, *P* < 0.05) (Fig. 2f). These in vitro and in vivo data suggest that Hypo-Exo exerts better cardioprotective effects against myocardial injury than Nor-Exo.

### LncRNA-UCA1 was a key component of Hypo-Exo-induced cardioprotection

**Fig. 3:**
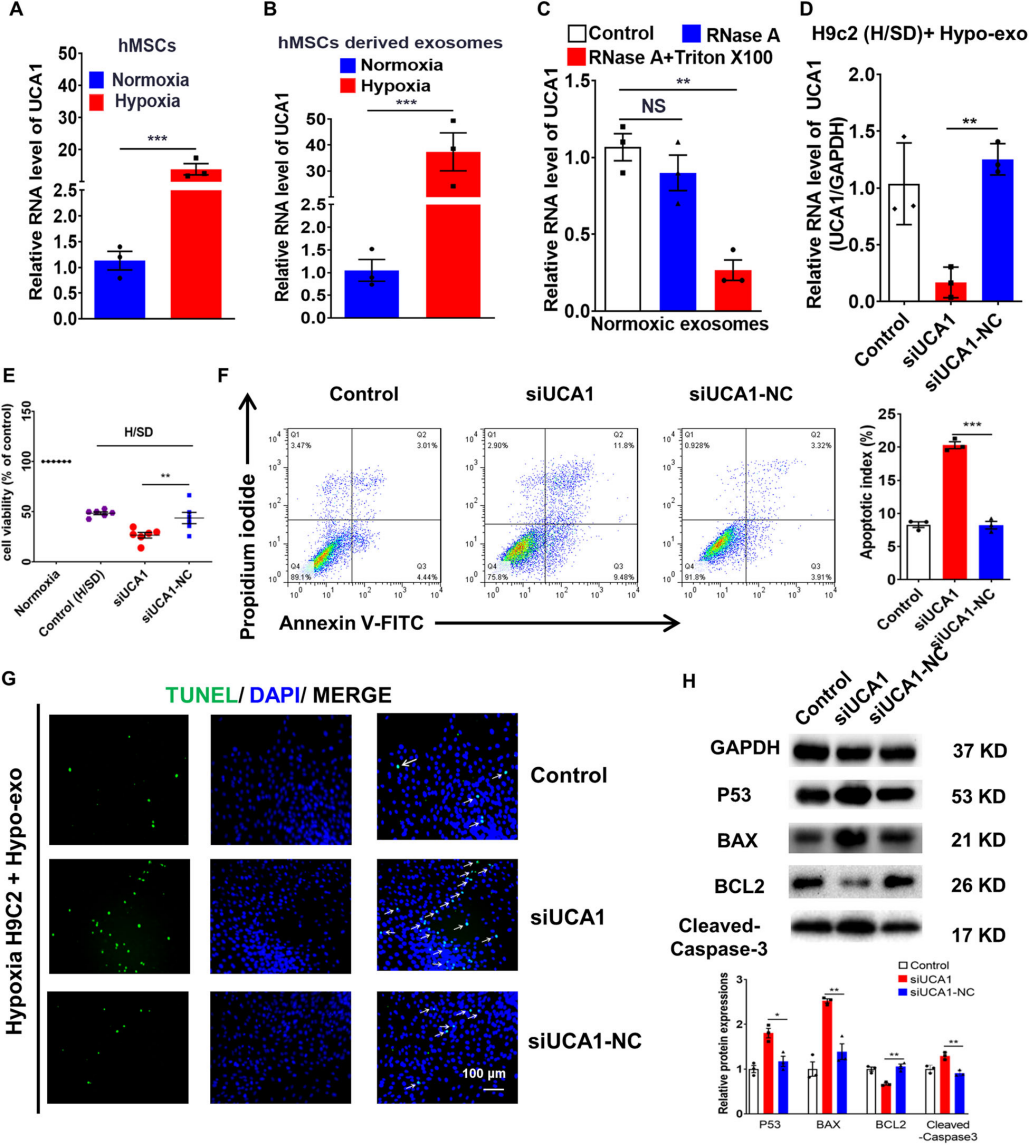
LncRNA-UCA1 was a key component in Hypo-Exo-induced cardioprotection in vitro. **a**, **b** Quantitative real-time PCR (qRT-PCR) analysis of lncRNA-UCA1 level in normoxic and hypoxic hMSCs (**a**) and exosomes (**b**) derived from hMSCs (*n* = 3). **c** qRT-PCR analysis of the lncRNA-UCA1 level in hypoxic exosomes derived from hMSCs. The samples were untreated or treated with RNase A (10 μg/ml) and/or 0.3% Triton X-100 and then further mixed with RNase inhibitor (*n* = 3). **d** Silencing UCA1 reduced the expression of lncRNA-UCA1 in hypoxic and ischemic H9c2 cells treated with Hypo-Exo (*n* = 3). **e**–**g** Hypoxic and ischemic H9c2 cells treated with Hypo-Exo were transfected with siRNA of UCA1 (siUCA1) and siRNA negative control (siUCA1-NC). SiUCA1 reduced cell viability by Cell Counting Kit-8 (CCK-8) (*n* = 6) (**e**) and promoted apoptosis by flow cytometric analysis (*n* = 3) (**f**) and TUNEL analysis (**g**). Green, TUNEL-positive nuclei; blue, DAPI-stained nuclei. White arrows indicate apoptotic cells. Scale bars, 100 μm. **h** Western blot analyzed p53, BAX, BCL-2, and cleaved-caspase-3 protein levels in hypoxic and ischemic H9c2 cells. Relative protein levels are presented as the average expression normalized to GAPDH. siUCA1 increased the expression of p53 and BAX, while the expression of BCL-2 was reduced (*n* = 3). Data are presented as mean ± SEM. Statistical analysis was performed with one-way ANOVA followed by Bonferroni’s correction. *P < 0.05, **P < 0.01, ***P < 0.001, NS not significant.

To clarify whether lncRNA-UCA1 in Exo was induced by hypoxia, intracellular and exosomal lncRNA-UCA1 levels was detected using qRT-PCR. Hypoxia induced upregulation of lncRNA-UCA1 not only in hMSCs but also in hMSC-derived Exo (Fig. 3a, b). The level of lncRNA-UCA1 significantly decreased in hypoxic Exo treated with both RNase A and Triton X-100, not just RNase A, suggesting that lncRNA-UCA1 exists in Exo (Fig. 3c). H9c2 cells were then co-cultured with Hypo-Exo under H/SD conditions. UCA1 silencer (si-UCA1 group) and its negative control (si-UCA1 NC group) were transfected into H9c2 cells. Inhibition of lncRNA-UCA1 attenuated the protective effects of Hypo-Exo in vitro (Fig. 3d–g). We then detected apoptosis-related proteins and found that protein expression of P53, BAX, and cleaved-caspase-3 significantly increased and the protein level of BCL-2 decreased remarkably in the siUCA1 group (Fig. 3h).

**Fig. 4:**
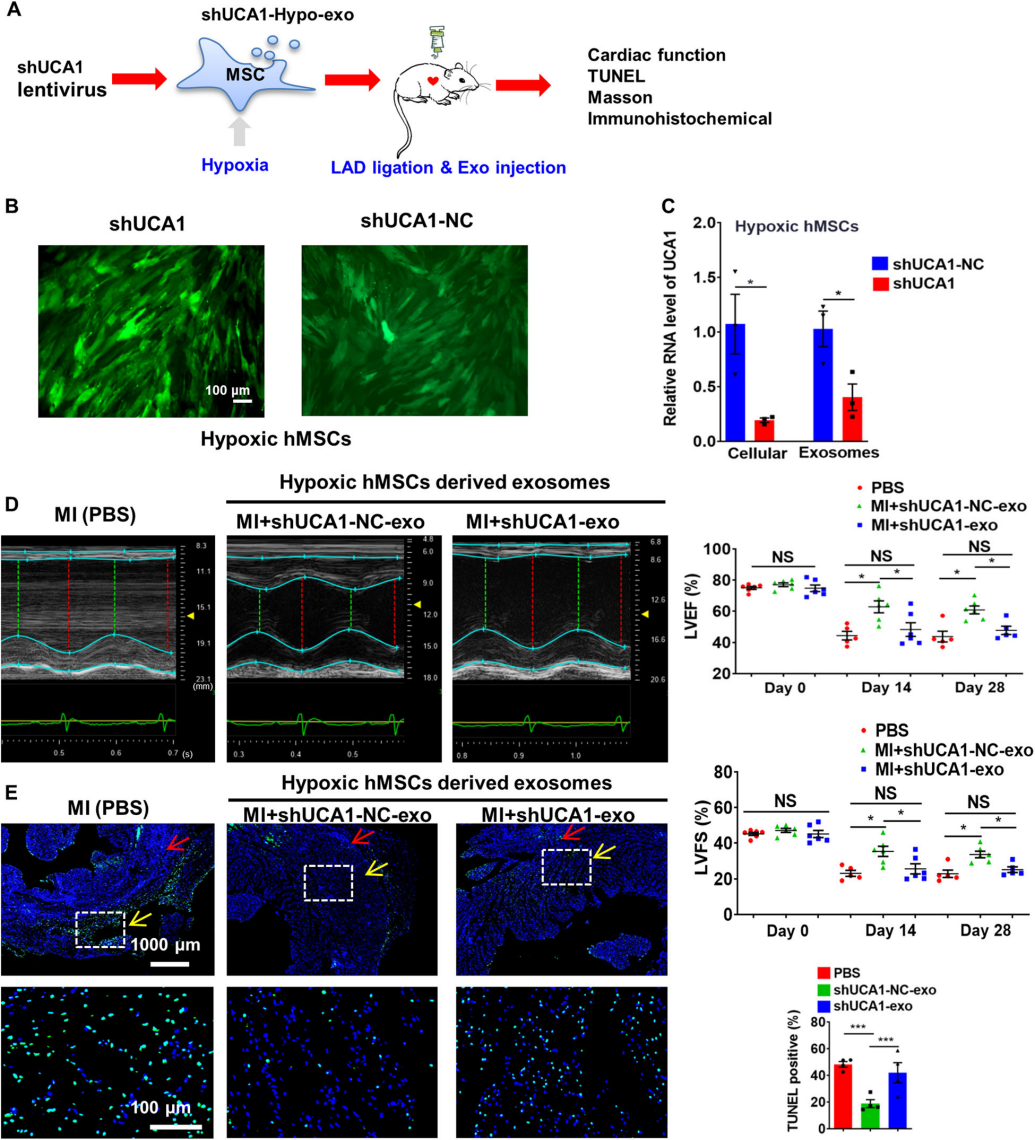
Effect of shUCA1-Exo on cardiac functional recovery of the infracted hearts. **a** The flowchart of in vivo experimental design. **b** Representative image shows the fluorescence (green) of hMSCs after transfection of shRNA vector or shRNA negative control. **c** qRT-PCR analyzed the lncRNA-UCA1 level in hMSCs and hMSC-derived exosomes. Data are normalized to spiked U6 and cel-miR-39 (*n* = 3). **d** Left ventricular ejection fraction (LVEF) and left ventricular fractional shortening (LVFS) measured by echocardiography at baseline (*n* = 6). Two weeks post-MI: PBS group (MI group, *n* = 5), shUCA1-NC-Exo group (*n* = 6), and shUCA1-Exo group (*n* = 6); 4 weeks post-MI: PBS group (MI group, *n* = 5), shUCA1-NC-Exo group (*n* = 6), and shUCA1-Exo group (*n* = 5). **e** Representative photographs showing the TUNEL-positive cells in the heart tissue among the different groups. Quantitative analysis of the apoptotic rate at the border zone among the different groups (4 random fields per animal; *n* = 4). Red arrows indicate the infarct zone and yellow arrows indicate the border zone. Data are presented as mean ± SEM. Statistical analysis was performed with one-way ANOVA followed by Bonferroni’s correction. *P < 0.05, ***P < 0.001, NS not significant.

To gain mechanistic insight into the role of exosomal lncRNA-UCA1 in Hypo-Exo-induced cardioprotection in the rat MI model, lncRNA-UCA1 loss-of-function studies were performed in Hypo-hMSCs (Fig. 4a). Here an shRNA vector (shUCA1-Exo group) or shRNA negative control (shUCA1-NC-Exo group) was transfected into Hypo-hMSCs; green fluorescence was observed (Fig. 4b). Then Exo were isolated. In addition, qPCR analysis confirmed that shUCA1-Exo significantly reduced cellular and exosomal lncRNA-UCA1 level compared with shUCA1-NC-Exo (Fig. 4c), indicating successful transfection. LVEF and LVFS were significantly decreased in shUCA1-Exo-injected rats compared with shUCA1-NC-Exo-injected rats at the 14th and 28th day after MI (Fig. 4d). Figure 4e shows significantly increased apoptotic cells at the border zone in shUCA1-Exo-treated rats compared with shUCA1-NC-Exo-treated rats (41.95% ± 16.60% vs. 18.82% ± 8.66%, *P* < 0.001).

**Fig. 5:**
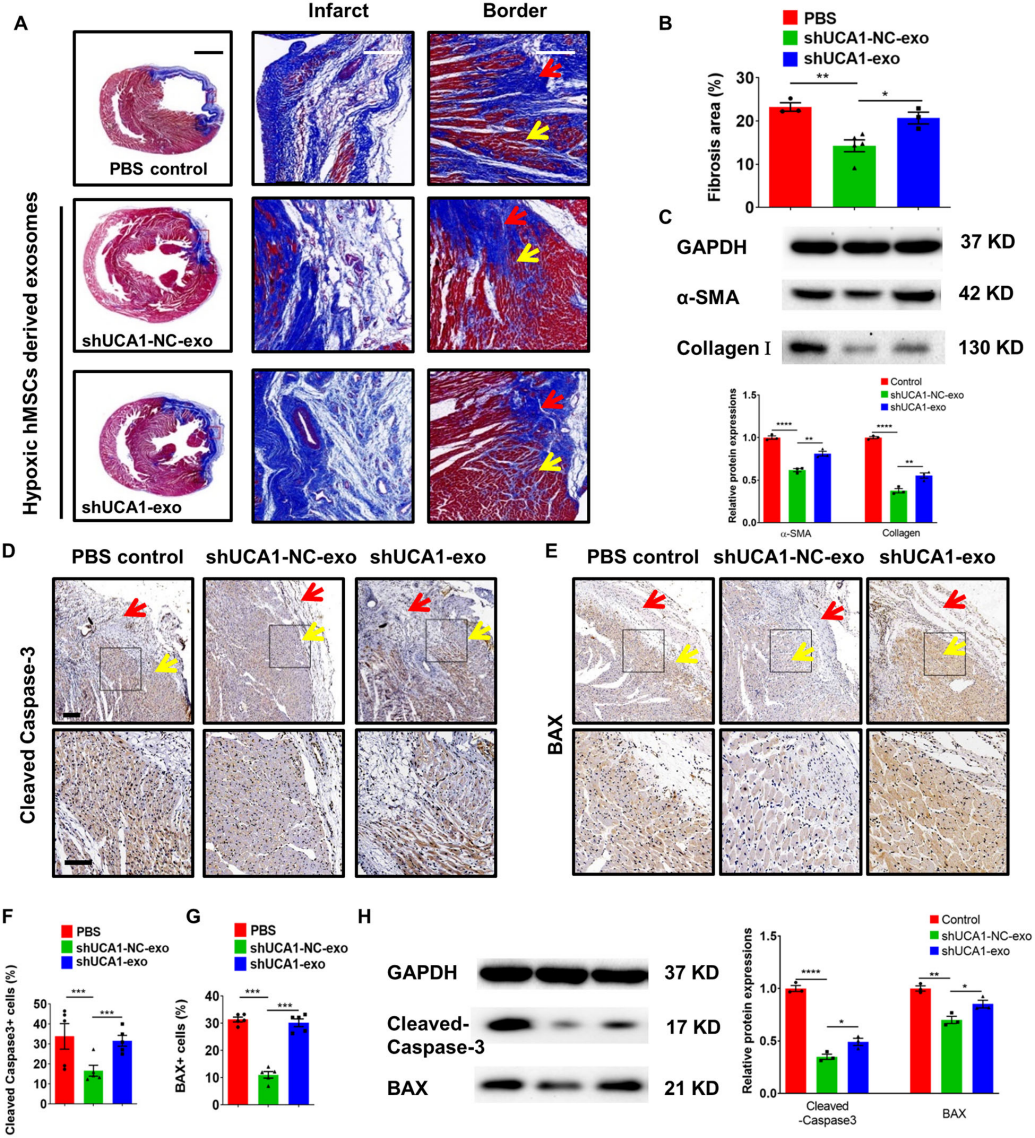
Effect of shUCA1-NC-Exo in the infracted hearts. **a** The representative Masson’s trichrome-stained myocardial cross-sections 4 weeks after AMI with the injection of PBS (MI group), shUCA1-Exo, and shUCA1-NC-Exo. Red arrows indicate the infarct zone and yellow arrows indicate the border zone. Scale bar, 2000 μm. Scale bar, 200 μm. **b** Quantification of fibrosis size among the different groups (*n* = 3, 5, and 3 for the PBS, shUCA1-NC-Exo, and shUCA1-Exo groups, respectively). **c** Representative western blot images and quantified data showing α-SMA and Collagen Ι protein level (*n* = 3). **d**, **e** Cleaved-caspase-3 (**d**) and BAX (**e**) staining at the border zone 28 days after MI. Red arrows indicate the infarct zone and yellow arrows indicate the border zone. **f**, **g** Quantitative analysis of cleaved-caspase-3^+^ (**f**) or BAX^+^ cells (**g**) at the border zone among the different groups (3 random fields per animal, *n* = 5). Scale bar = 200 μm (above), 100 μm (below). **h** Representative western blot images and quantified data showing cleaved-caspase-3 or BAX protein level (*n* = 3). Data are presented as mean ± SEM. Statistical analysis was performed with one-way ANOVA followed by Bonferroni’s correction. *P < 0.05, **P < 0.01, ***P < 0.001, ****P < 0.0001.

Quantification of fibrosis size of the hearts 4 weeks post-MI showed that shUCA1-NC-Exo-injected rats had a reduced size compared with shUCA1-Exo- and PBS-injected rats (Fig. 5a, b). As shown in Fig. 5c, shUCA1-Exo injection also increased the expression of α-smooth muscle actin and Collagen Ι compared with shUCA1-NC-Exo injection after MI. The number of cleaved-caspase-3^+^ and BAX^+^ cells was also significantly lower in the shUCA1-NC-Exo group than in the shUCA1-Exo group (Fig. 5d–g). Similar trends were revealed by western blot analysis (Fig. 5h). These results show that lncRNA-UCA1 is a key component of the cardioprotection afforded by Hypo-Exo in vitro and in vivo.

### LncRNA-UCA1 exerted cardioprotective effects by sponging miR-873-5p

**Fig. 6:**
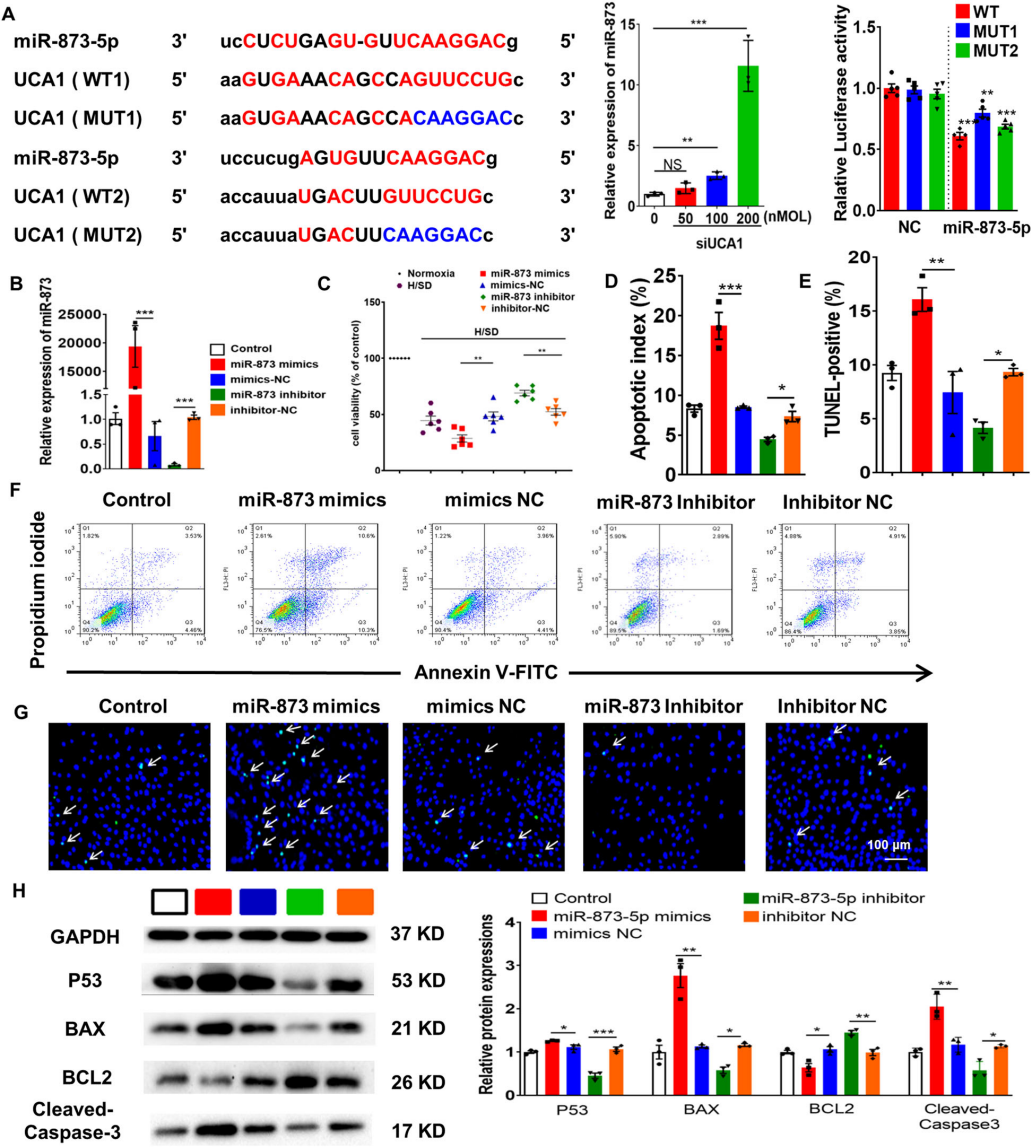
LncRNA-UCA1 exerted cardioprotective effects by sponging miR-873-5p. **a** The putative binding sites for miR-873-5p in the 3’UTR of lncRNA-UCA1. Relative expression of miR-873-5p in hypoxic and ischcemic H9c2 cells transfected with UCA1 smart silencer (*n* = 3). Data are normalized to U6. Luciferase activity assay of HEK-293T cells transfected with luciferase constructs containing WT-3'UTR and MUT-3'UTR of lncRNA-UCA1 (*n* = 5). **b** Cells were transfected with miR-873-5p mimics, miR-873-5p inhibitor, and corresponding scrambled control. Relative miR-873-5p expression is presented as the average expression normalized to U6 (*n* = 3). **c**–**g** Hypoxic and ischemic H9c2 cells were transfected with miR-873-5p mimics, miR-873-5p inhibitor, and corresponding scrambled controls. Cell viability was assessed by CCK8 (*n* = 6) (**c**). Cell apoptosis was analyzed by flow cytometric analysis (*n* = 3) (**f**, **d**) and TUNEL assay (*n* = 3) (**g**, **e**). Green, TUNEL-positive nuclei; Blue, DAPI-stained nuclei. White arrows indicate apoptotic cells. Scale bars, 50 μm. **h** Hypoxic and ischemic H9c2 cells were transfected with miR-873-5p mimics, inhibitors, and both negative controls, and western blot used to analyze p53, BAX, BCL-2, and cleaved-caspase-3 protein levels in H9c2 cells. Relative protein levels are presented as the average expression normalized to GAPDH (*n* = 3). Data are presented as mean ± SEM. Statistical analysis was performed with one-way ANOVA followed by Bonferroni’s correction. *P < 0.05, **P < 0.01, ***P < 0.001.

In hypoxic and ischemic cardiomyocytes treated with Hypo-Exos, qRT-PCR revealed that inhibition of lncRNA-UCA1 significantly elevated miR-873-5p in a dose-dependent manner (Fig. 6a). The binding sites for lncRNA-UCA1 and miR-873-5p were examined using a luciferase reporter assay. We cloned either wild-type 3'UTR or mutant 3′UTRs of lncRNA-UCA1 in the putative miR-873-5p-binding sites into a reporter plasmid and assessed their responsiveness to miR-873-5p in 293T cells. The results showed that miR-873-5p reduced luciferase activity for lncRNA-UCA1 wild-type 3′UTR constructs. When one of the binding sites was mutated, luciferase activity was also reduced. These results show that lncRNA-UCA1 binds to both the binding sites (Fig. 6a).

H9c2 cells under H/SD conditions were then transfected with the miR-873-5p mimics and inhibitor (Fig. 6b). The miR-873-5p inhibitor promoted significant cardioprotective effects that were attenuated by miR-873 mimics (Fig. 6c–g). Moreover, if hypoxic or ischemic H9c2 cardiomyocytes were transfected with a miR-873-5p mimic, there was significant upregulation of BAX, P53, and cleaved-caspase-3 and downregulation of BCL-2 (Fig. 6f). These data demonstrate that lncRNA-UCA1 contains functional miR-873-5p-binding sites and acts as a sponge for miR-873-5p.

### miR-873 enhanced the phosphorylation of AMPK in cardiomyocytes by targeting XIAP

**Fig. 7:**
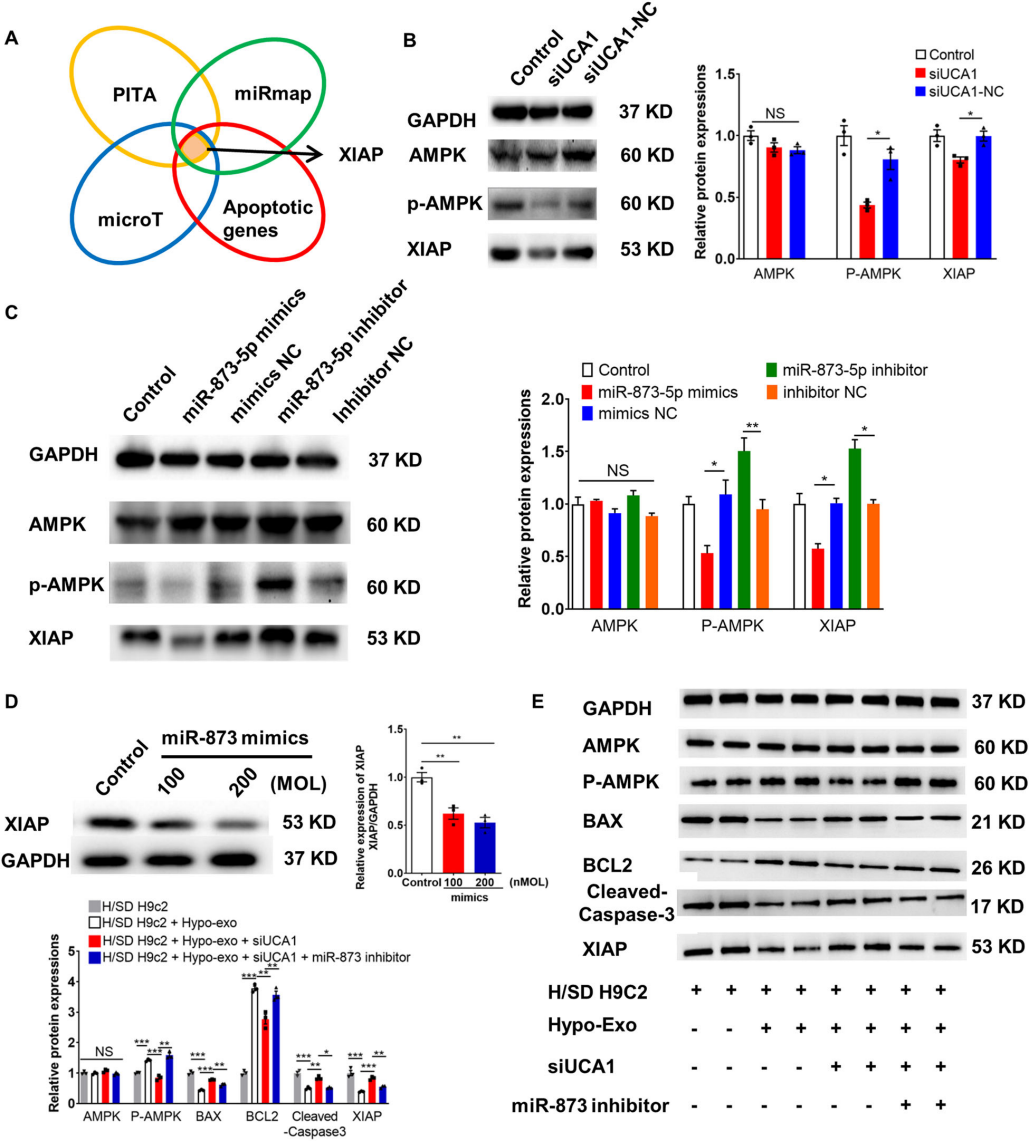
miR-873 enhanced the phosphorylation of AMPK in cardiomyocytes by targeting XIAP. **a** Screening scheme for putative target genes that might contribute to the antiapoptotic effects. **b** Representative western blot and quantified data show expression of XIAP and phosphorylation of AMPK in hypoxic and ischemic H9c2 cells treated with Hypo-Exo (*n* = 3). **c** Representative western blot and quantified data show XIAP and phosphorylation of AMPK in H9c2 cells treated with a miR-873-5p mimic, mimics negative control (NC), miR-873-5p inhibitor, or inhibitor NC (*n* = 3). **d** H9c2 cells were transfected with a miR-873-5p mimic, mimics negative control (NC), miR-873-5p inhibitor, or inhibitor NC. The XIAP level was analyzed by western blot (*n* = 3). **e** Representative western blot and quantified data show the expression of the indicated proteins in H9c2 cells (*n* = 3). Data are presented as mean ± SEM. Statistical analysis was performed with one-way ANOVA followed by Bonferroni’s correction. *P < 0.05, **P < 0.01, ***P < 0.001, NS not significant.

The target genes of miR-873-5p were predicted by PITA (http://genie.weizmann.ac.il/pubs/mir07/mir07_data.html)^19^, miRmap (http://mirmap.ezlab.org)^20^, and Diana-microT (http://diana.imis.athena-innovation.gr/DianaTools/index.php?r=microT_CDS/index)^21,22^. Among the possible miR-873-5p target genes that are also involved in the apoptosis pathway, we focused on XIAP (Fig. 7a). Western blot analysis showed that silencing lncRNA-UCA1 suppressed the protein level of XIAP and phosphorylation of AMPK (Fig. 7b). In addition, miR-873-5p mimics reduced the phosphorylation of AMPK and XIAP level (Fig. 7c). Transfection of miR-873 mimics was associated with a decreased protein level of XIAP (Fig. 7d). Although silencing UCA1 could increase the protein level of BAX and downregulate the protein level of BCL-2 and XIAP and phosphorylation of AMPK in hypoxic H9c2 cells treated with hypoxic Exo, transfection of the miR-873 inhibitor could rescue these changes (Fig. 7e). These data suggest the essential role of the UCA1/miR-873-5p/XIAP axis in cardioprotection.

### Circulating exosomal lncRNA-UCA1 may be a promising novel biomarker for AMI diagnosis

**Fig. 8:**
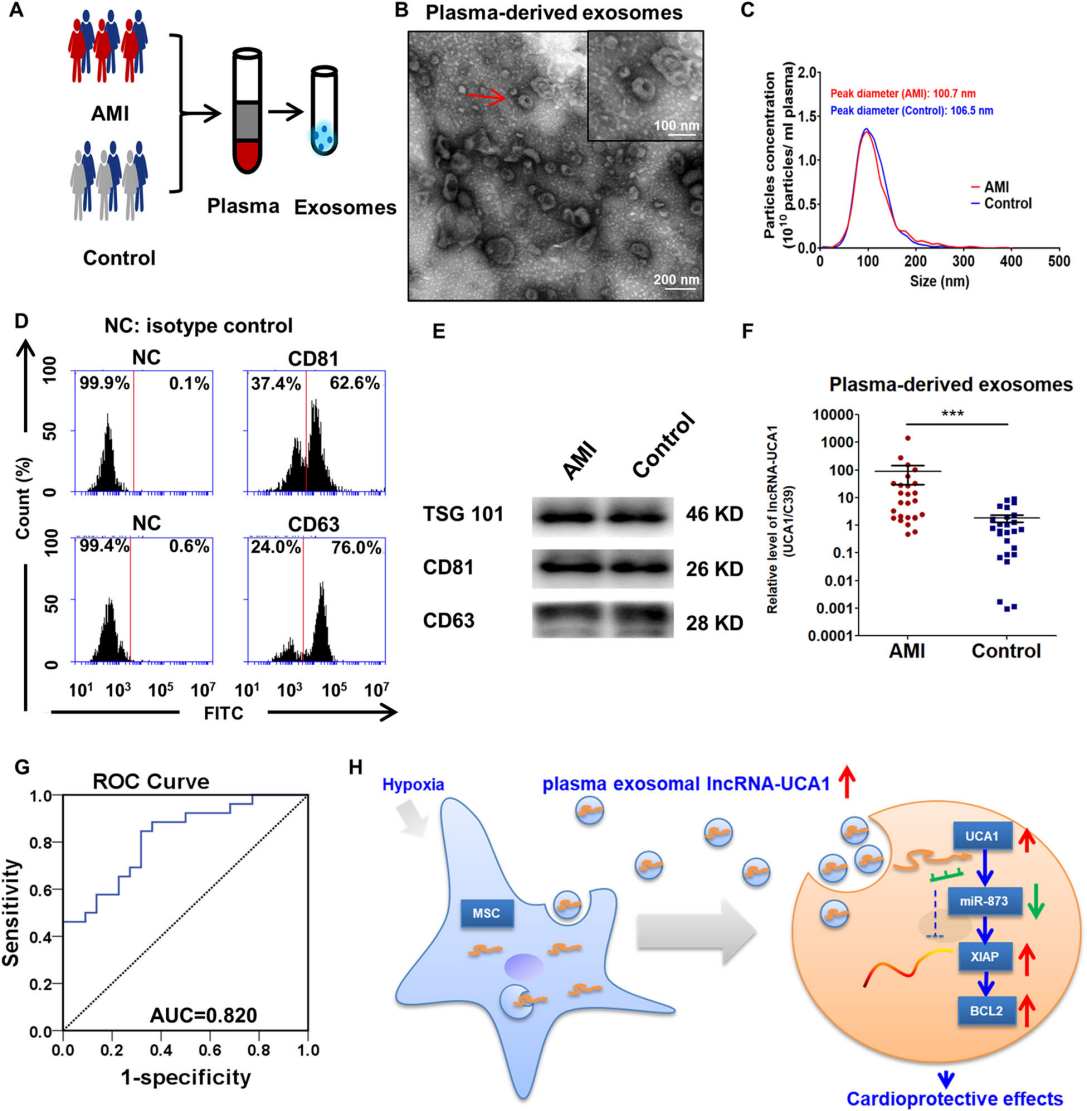
Circulating exosomal lncRNA-UCA1 may be a promising novel biomarker for AMI diagnosis. **a** Exosomes were isolated from the plasma of patients with AMI within 12 h or from healthy volunteers. **b** Representative electron micrograph of the extracted exosomes. Red arrows indicate plasma-derived exosomes. Scar bar: 100 nm. **c** Nanoparticle tracking analysis demonstrating size distribution of exosomes purified from AMI patients and healthy volunteers. *X* axis represents size of exosomes. *Y* axis represents the particle concentration in 1 ml PBS (before dilution). **d** Surface markers (CD63, CD81) of plasma exosomes were detected by flow cytometry. Exosomes that were not stained with CD63 or CD81 antibodies were labeled as NC group. **e** Western blots showing the protein levels of TSG101, CD63, and CD81 in plasma-derived Exo. **f** qRT-PCR analysis of UCA1 level in plasma exosomes from AMI patients (*n* = 26) and healthy volunteers (*n* = 26). Data are normalized to spiked cel-miR-39 (C39). Data are presented as mean ± SEM. ***P < 0.001. Statistical analysis was performed with Mann–Whitney *U* test. **g** The ROC curve for the diagnostic value of plasma-derived exosomal lncRNA-UCA1. (*n* = 52). **h** Exosomal lncRNA-UCA1 derived from hypoxic hMSCs promoted cell viability and attenuated apoptosis via miR-873-5p/XIAP/p-AMPK axis. Moreover, exosomal lncRNA-UCA1 in plasma may serve as a potential noninvasive biomarker for the diagnosis of acute myocardial infarction (AMI).

Patients with AMI (*n* = 26) and healthy donors (*n* = 26) were enrolled. The clinical characteristics of the enrolled patients are listed in Supplementary Table 4. Exo were extracted from the plasma of AMI patients and healthy donors (Fig. 8a). TEM analysis revealed that the average size of these isolated extracellular vesicles was consistent with Exo (Fig. 8b). NTA confirmed no significant differences in size distribution between the two groups (Fig. 8c). No significant differences were found in average size or concentrations between plasma-derived Exo from AMI and controls (Supplementary Table 5). Flow cytometric analysis showed that the circulating Exo were positively expressed for exosomal protein markers, such as CD81 and CD63 (Fig. 8d). Western blot analysis revealed that circulating Exo were positive for the exosome-specific markers TSG101, CD81, and CD63 (Fig. 8e). We also found that the level of exosomal lncRNA-UCA1 was markedly higher in AMI patients than in healthy volunteers (Fig. 8f). The ROC analysis indicated that circulating exosomal lncRNA-UCA1 (AUC = 0.82) might be a novel diagnostic biomarker of AMI (Fig. 8g). Collectively, these data demonstrate that circulating exosomal lncRNA-UCA1 may be a promising novel biomarker for AMI diagnosis.

## Discussion

There are three major findings of the current study. First, hypoxia hMSC-derived Exo transmit protective signals and endow cardioprotective effects to prevent MI injury. Second, we identified hMSC-derived exosomal lncRNA-UCA1 as a major cardioprotective molecule in the ischemic heart. Third, circulating exosomal lncRNA-UCA1 may serve as a noninvasive biomarker for AMI diagnosis (Fig. 8h). These findings reveal a novel mechanism by which hypoxic hMSC-derived Exo exert cardioprotective effects through the exosomal lncRNA-UCA1/miR873-5p/XIAP axis.

Noncoding RNAs encapsulated in Exo are particularly stable in body fluids because the RNAs are free from degradation by ribonuclease^23^. We confirmed that Exo provided a protective membrane for lncRNA-UCA1 against RNase degradation. Exo are released from cardiomyocytes and enter body fluids^24^, including plasma, serum, and urine. The level of some exosomal lncRNAs is associated with the prognosis of cardiovascular diseases and may be useful as noninvasive biomarkers^25,26^. The present study showed that exosomal lncRNA-UCA1 in AMI patients is much higher than in normal controls. These results provide novel evidence that circulating exosomal lncRNA-UCA1 may serve as a potential biomarker for the diagnosis of AMI.

Previous studies have shown that the plasma level of UCA1 decreases 2 h after AMI and reaches its lowest point after 6–12 h. After 48–72 h of AMI onset, the circulating UCA1 level begins to recover and is restored to the control level. UCA1 level has been shown to be higher 72–96 h after AMI than in non-AMI subjects^27^, contrary to the results of the present study. The reason is that lncRNA was detected in plasma, whereas in the current study lncRNA was detected in plasma-derived Exo. Tian et al.^28^ found that the level of two onco-miRNAs (miR-181b-5p and miR-21-5p) was significantly higher in Exo than in plasma in lung cancer patients. Xie et al.^29^ performed miRNA profiling using plasma and plasma-derived Exo samples from two animal models of kidney disease and revealed that, because of the differential changes in miRNAs, measurement of exosomal miRNAs could not be replaced by measurement of miRNA in plasma or vice versa. We infer that plasma lncRNA level may differ from plasma-derived exosomal lncRNA level. Yu et al.^30^ reported that plasma lncRNA UCA1 may be a good indicator for the diagnosis of chronic heart failure (CHF) and might predict poor CHF outcomes. Taken together, these studies have proposed lncRNA-UCA1 as a potential biomarker in the cardiovascular system.

Liu et al.^31^ reported that a decrease in UCA1 level promoted apoptosis of primary cardiomyocytes by stimulating expression of the p27 protein. Wang et al.^32^ reported that inhibition of miR-143 resulted in a protective effect of lncRNA UCA1 on cardiomyocytes against hypoxia/reoxygenation-induced apoptosis. Our study revealed these cardiac-protective effects via exosome-derived lncRNA-UCA1 in both in vivo and in vitro experiments. These results are consistent with the observation of UCA1 expression and apoptosis following cardiac injury, suggesting that UCA1 might play an important role in the process of ischemia and hypoxia of cardiomyocytes.

Compared with liposomes and polyethylenimine, local injection of miR21-enriched extracellular vesicles from cells has demonstrated therapeutic efficacy in a mouse model of MI^33^. Thus cell-derived Exo may be a valuable clinical alternative for MI treatment. It has been reported that the intramyocardial delivery of Hypo-Exo in mice can reduce infarct size and enhance cardiac repair following MI, and miR-125b has been identified as an exerkine with protective antiapoptotic effects^13^. Nonetheless, other noncoding RNAs may also contribute to these antiapoptotic and therapeutic effects. In our study, we speculate that hypoxic hMSCs promoted lncRNA-UCA1 transferred in Exo to mediate the phenotypes for cardiomyocytes. Our results are consistent with this hypothesis. Hypoxia promotes the secretion of lncRNA-UCA1-enriched Exo from hMSCs, which play a cardioprotective role in cardiomyocytes. In addition, our study demonstrates that lncRNA-UCA1 regulates apoptosis via the miRNA-873-5p/XIAP axis. Both XIAP and BCL-2 were considered antiapoptotic genes, and it was also reported that XIAP could serve as an E3 ligase for BCL-2; thus degradation of BCL-2 by XIAP could promote apoptosis^34^.

The present study has some limitations. First, H9c2 cells, which are known to undergo expansive oxidative stress with further passaging^35^, are not a good representation of cardiomyocytes. Primary neonatal rat cardiomyocytes are a more preferable source for in vitro studies. Nonetheless, it is also reported that H9c2 cells and primary neonatal cardiomyocyte cells show similar hypertrophic responses in vitro^36^. Second, although intramyocardial injection is an efficient means by which to accurately deliver cardiac gene to the target area, this strategy causes damage and the injected Exo may leak at the injection site. Third, because of the lack of targeting, these Exo may also be absorbed by other cell types of the heart, leading to extra-target effects. Gene or chemical modification of extracellular vesicles may be an effective way to solve this problem. Finally, the identification of the origin of circulating Exo needs further elucidation because of the inadequate understanding of the sorting processes in determining which RNA species are packaged into Exo and the fact that exosomal RNA content does not merely parrot the RNA profile of the secreting cell.

In summary, the present study has demonstrated that hypoxia promotes hMSCs to secret lncRNA-UCA1-enriched Exo that may play a cardioprotective role in cardiac injury through the lncRNA-UCA1/miR873-5p/XIAP axis, and exosomal lncRNA-UCA1 in human plasma may be considered a potential noninvasive biomarker for the diagnosis of AMI.

## Supplementary information

Supplementary information

Supplementary information2

Supplementary information3

Supplementary information4
