# Supplementary material for: Long noncoding RNA UCA1 from hypoxia-conditioned hMSC-derived exosomes: a novel molecular target for cardioprotection through miR-873-5p/XIAP axis
Source: Cell Death Dis. 2020 Aug 10;11(8):696. doi: 10.1038/s41419-020-02783-5 (PMC7442657; doi:10.1038/s41419-020-02783-5)
Supplement: Supplementary file 1 — Supplementary information [file 41419_2020_2783_MOESM1_ESM.docx]

**Supplementary table legends**

**Supplementary Table 1. Sequences of primer, UCA1 silencer, miR-873-5p mimics, inhibitor and their negative controls**

**Supplementary Table 2. The three shRNA sequences list**

**Supplementary Table 3. Particle concentration, size and protein concentration of hMSCs-derived exosomes**

**Supplementary Table 4. Baseline characteristics**

**Supplementary Table 5. Particle concentration, size and protein concentration of plasma-derived exosomes**
