# Supplementary material for: Long noncoding RNA UCA1 from hypoxia-conditioned hMSC-derived exosomes: a novel molecular target for cardioprotection through miR-873-5p/XIAP axis
Source: Cell Death Dis. 2020 Aug 10;11(8):696. doi: 10.1038/s41419-020-02783-5 (PMC7442657; doi:10.1038/s41419-020-02783-5)
Supplement: Supplementary file 2 — Supplementary information2 [file 41419_2020_2783_MOESM2_ESM.docx]

**Supplementary table 1.**

| **Primers** | **Sequences (5'–3')** |
| --- | --- |
| **Primers for quantitative real-time PCR or RT-PCR** | |
| UCA1-F | CTCAAACTCTACAGCCTCAATGG |
| UCA1-R | CATGGCTTTATTCTGGAATGGT |
| Human miR-873-5p-F | GGGGCAGGAACTTGTGAG |
| miR-873-5p-R | GTGTGGTGTGGTATGGTGTG |
| Rat miR-873-5p-F | GGGGCAGGAACTTGTGAG |
| miR-873-5p-R | GTGTGGTGTGGTATGGTGTG |
| GAPDH-F | GAACGGGAAGCTCACTGG |
| GAPDH-R | GCCTGCTTCACCACCTTCT |
| U6-F | CTCGCTTCGGCAGCACA |
| U6-R | AACGCTTCACGAATTTGCGT |
| Cel-miR-39-3p F | GGGTCACCGGGTGTAAATC |
| Cel-miR-39-3p R | GAGAGGAGAGGAAGAGGGAA |
| **UCA1 silencing** | |
| UCA1 smart silencer-1 | CCATCAGATCCTTGCCCAT |
| UCA1 smart silencer-2 | GGTAATGTATCATCGGCTT |
| UCA1 smart silencer-3 | GCTAACTGGCACCTTGTTA |
| **miR-873-5p mimics, inhibitor** | |
| miR-873-5p mimics | GCAGGAACUUGUGAGUCUCCU AGGAGACUCACAAGUUCCUGC |
| miR-873-5p mimics negative control | UUUGUACUACACAAAAGUACUG |
|  | CAGUACUUUUGUGUAGUACAAA |
| miR-873-5p inhibitor | AGGAGACUCACAAGUUCCUGC |
| miR-873-5p inhibitor negative control | CAGUACUUUUGUGUAGUACAAA |

**Supplementary table 2.**

| **UCA1 shRNA and Negetive control** | **Sequences (5'–3')** |
| --- | --- |
| LV-UCA1-RNAi negative control | TTCTCCGAACGTGTCACGT |
| LV-UCA1-RNAi-1 | GCTGAGGATAACCACCTTT |
| LV-UCA1-RNAi-2 | GGACAACACAAAGTATGTT |
| LV-UCA1-RNAi-3 | CCACCTACATTAAAGCTAA |

**Supplementary table 3.**

|  | Nor-exo (n=3) | Hypo-exo (n=3) | P value |
| --- | --- | --- | --- |
| **BCA protein assay** |  |  |  |
| protein concentration (μg/μl) | 1.07± 0.09 | 1.49± 0.08 | 0.004^**^ |
| **NTA** |  |  |  |
| concentration  (10^9^ Particles/ml) | 3.83± 0.21 | 60.00± 17.32 | < 0.001^***^ |
| size (nm) | 115.93± 2.71 | 109.53± 7.88 | 0.254 |
| **TEM** |  |  |  |
| size (nm) | 118.62± 7.64 | 110.22± 12.98 | 0.202 |
| Data are described using mean ± standard deviation (SD); NTA: nanoparticle tracking analysis; TEM: transmission electron microscopy. **P < 0.01, ***P < 0.001 | | | |

**Supplementary table 4.**

| Characteristics | Normal (n=26) | AMI (n=26) | P value |
| --- | --- | --- | --- |
|  |  |  |  |
|  |  |  |  |
| Age, y | 64.92 ± 15.21 | 68.81 ± 13.46 | 0.33 |
| Male, n% | 22 (84.6%) | 16 (61.54%) | 0.06 |
| Body mass index, Kg/m^2^ | 24.24 ± 2.40 | 23.75 ± 3.03 | 0.54 |
| Hypertension, n% | 21 (80.77%) | 18 (69.23%) | 0.34 |
| Diabetes, n% | 3 (11.54%) | 6 (23.08%) | 0.27 |
| Current or former smoker, n% | 13 (50.00%) | 10 (38.46%) | 0.40 |
| Alcohol consumption, n% | 5 (19.23%) | 3 (11.54%) | 0.70 |
| LVEF, % | 59.90 ± 5.23 | 52.06 ± 8.40 | <0.01 |
| SBP, mmHg | 129.54 ± 22.51 | 130.81 ± 28.67 | 0.86 |
| DBP, mmHg | 77.15 ± 12.60 | 76.54 ± 20.24 | 0.90 |
| Heart rate, bpm | 81.04 ± 17.82 | 90.46 ± 22.79 | 0.10 |
| WBC, 10^9^/L | 7.81 ± 1.47 | 12.18 ± 3.10 | <0.001 |
| Hemoglobin, g/L | 139.00 ± 22.48 | 138.88 ± 18.04 | 0.98 |
| Blood glucose, mmol/L | 8.44 ± 3.92 | 10.47 ± 5.50 | 0.13 |

|  | AMI (n=3) | Control (n=3) | P value |
| --- | --- | --- | --- |
| **BCA protein assay** |  |  |  |
| protein concentration (μg/μl) | 6.47± 1.06 | 6.13± 0.59 | 0.651 |
| **NTA** |  |  |  |
| Concentration  (10^11^ Particles/ml) | 2.70± 0.61 | 2.30± 0.61 | 0.466 |
| size (nm) | 115.97± 3.41 | 110.67± 2.40 | 0.662 |
| **TEM** |  |  |  |
| size (nm) | 101.10± 13.29 | 107.26± 19.35 | 0.523 |
| Data are described using mean ± standard deviation (SD). NTA: nanoparticle tracking analysis; TEM: transmission electron microscopy. | | | |

**Supplementary table 5.**
