# Supplementary material for: Long noncoding RNA UCA1 from hypoxia-conditioned hMSC-derived exosomes: a novel molecular target for cardioprotection through miR-873-5p/XIAP axis
Source: Cell Death Dis. 2020 Aug 10;11(8):696. doi: 10.1038/s41419-020-02783-5 (PMC7442657; doi:10.1038/s41419-020-02783-5)
Supplement: Supplementary file 3 — Supplementary information3 [file 41419_2020_2783_MOESM3_ESM.pdf]

Supplementary Figure 1. (A) and (B) Representative western blot images and quantified data of BAX, BCL-2, P53 and cleaved-caspase3 protein levels in H9c2 cells (H/SD condition) treated with PBS, Nor-exo or Hypo-exo (n=3). Data are presented as mean ± SEM. Statistical analysis was performed with one-way ANOVA followed by Bonferroni’s correction. *$P < 0.05$.
