# Supplementary figures and images for: Long noncoding RNA UCA1 from hypoxia-conditioned hMSC-derived exosomes: a novel molecular target for cardioprotection through miR-873-5p/XIAP axis
Source: Cell Death Dis. 2020 Aug 10;11(8):696. doi: 10.1038/s41419-020-02783-5 (PMC7442657; doi:10.1038/s41419-020-02783-5)

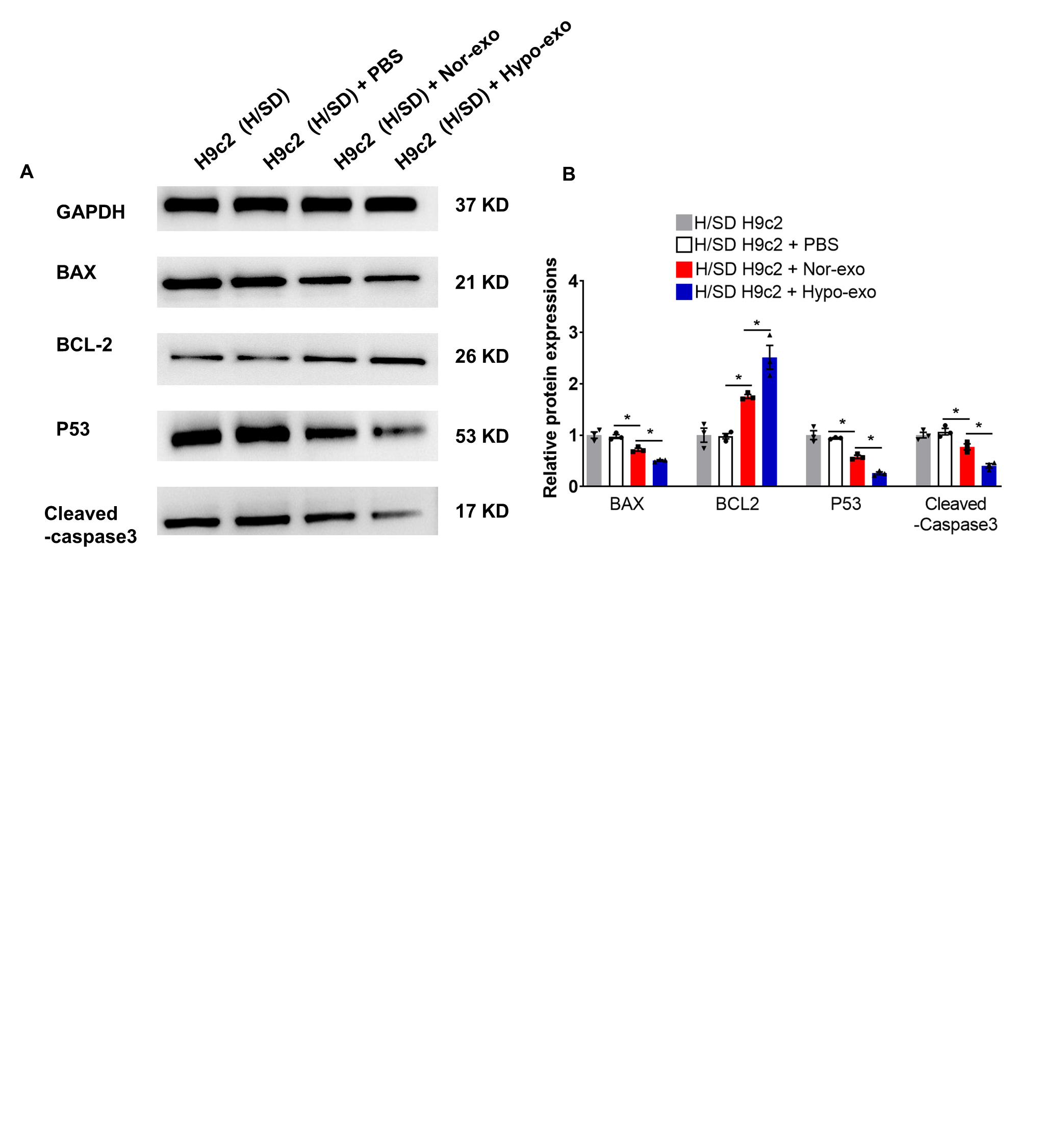

Supplement: Supplementary file 4 — Supplementary information4 [file 41419_2020_2783_MOESM4_ESM.tif]
